# Combination of TLR agonist and miR146a mimics attenuates ovalbumin-induced asthma

**DOI:** 10.1186/s10020-020-00191-1

**Published:** 2020-06-29

**Authors:** Xinhua Wang, Xiaoxian Lu, Chenhui Ma, Lihong Ma, Shuguang Han

**Affiliations:** 1 Department of Respiratory Medicine, The Affiliated Wuxi No.2 People’s Hospital of Nanjing Medical University, No.68 Zhongshan Road, Liangxi District, Wuxi, 214002 Jiangsu China; 2 Department of Critical Care Medicine, The Affiliated Wuxi No.2 People’s Hospital of Nanjing Medical University, No.68 Zhongshan Road, Liangxi District, Wuxi, 214002 Jiangsu China

**Keywords:** miR146a-mimic, Asthma, TLR2, ILC2, IL5

## Abstract

**Background:**

microRNA-146a has been reported to be a regulator in the process of attenuating asthma by inhibiting Toll-like receptor 2 (TLR2) pathway. This study aimed to investigate how miR146a-inhibitor affect the symptom of asthma and the underlying mechanisms.

**Methods:**

Ovalbumin (OVA)-induced allergic asthma mice model was established by intraperitoneal injection with 20 μg of OVA. Total cells and differential inflammatory cells in bronchoalveolar lavage fluid were counted by flow cytometry. The expression levels of molecules and cytokines in TLR2 signaling pathway were detected by Q-PCR and ELISA.

**Results:**

miR146a-inhibitor attenuated OVA-induced allergic asthma by increasing Th1 cytokines in OVA-induced allergic asthma model, and the treatment of miR146a-inhibitor can reduce the inflammation caused by asthma, followed by the down-regulation of IL-5 and IL-13 in sorted ILC2. The inhibition of miR-146a significantly reduced symptoms of asthma model with TLR2-related molecules being up-regulated.

**Conclusion:**

It was found that miR-146a is an important regulator in OVA-induced allergic asthma model, which can relieve symptoms of asthma through regulating TLR2 pathway. These findings provide a theoretical basis for solving asthma in clinical treatment.

## Introduction

Bronchial asthma (referred as asthma) is a relatively common chronic respiratory disease (Papi et al. 2018; Chung 2015). In recent years, the prevalence rate has increased year by year. The global prevalence of doctor-diagnosed asthma in adults is 4.3%, which brings a heavy economic and social burden to the world and becomes an increasingly serious medical and health problem. It is of great significance to understand its pathogenesis and find potential treatment (Schatz and Rosenwasser 2014; Rabe et al. 2018).

Allergic asthma is the most common type of asthma, resulting in a series of symptoms associated with allergen exposure, such as chest tightness, cough, and wheezing (Cohn et al. 2004). Evidences indicate that allergic asthma is attributed to the synergistical consequence of inflammatory cells (lymphocytes, eosinophils, mast cells, and macrophages), often characterized by increased biomarkers including eosinophils, serum IgE, and Th2 type cytokines (Schatz and Rosenwasser 2014; Gibson 2017). Th2 type cytokines (e.g. interleukin (IL)-4, IL-5, and IL-13) play a major role in the pathogenesis of allergic asthma (Chung 2015; Sui et al. 2018). Among them, IL-4 promotes the production of specific IgE, which binds to and activates the corresponding receptors on the surface of mast cells to release various inflammatory factors, leading to bronchial smooth muscle spasm and stenosis (Galli et al. 2008). IL-5 stands out in the growth, differentiation, recruitment and survival of eosinophils, which are crucial in asthma inflammation and airway remodeling. In addition, IL-13 plays a critic role in Th2-type responses such as eosinophilic inflammation, mucus hypersecretion, airway hyperactivity (AHR), and airway remodeling (Izuhara 2017; Sheridan 2018). Therefore, Th2-type cytokines have become a hotspot of allergic asthma research. In recent years, with the further study of asthma, the role of other immune cells (such as macrophages, inflammatory monocytes, etc.) in allergic asthma has gradually been revealed, while that of eosinophils hasn’t been well illustrated.

Previous studies demonstrate that miR146a mimics are able to inhibit the function and proliferation of type II innate lymphoid cells (ILC2) (Kim et al. 2009; Li et al. 2014; Stickel et al. 2017), thereby alleviating the symptoms of asthma to some extent (Etikala et al. 2015; Hayakawa and Wang 2018), while this effect of miR146a is not so satisfying. Excitedly, several studies further indicated that activation of Toll-like receptors (TLRs) can effectively relieve asthma symptoms (West et al. 2010), and miR146a is upregulated by and negatively regulates TLR2 pathway in bacterial stimulation (Alderton 2012; He et al. 2014). Hence, we propose it is possible to achieve better healing by combining TLR2 agonists with miR146 modulating agents.

## Methods and materials

### Animals

Specific pathogen-free C57BL/6 mice at the age of 8 weeks old each weighing 20 to 22 g were housed with food and water provided ad libitum under a 12 h light/dark cycle. On days 0, 7, and 14, the mice were sensitized by intraperitoneal injection with 20 μg of ovalbumin (OVA) emulsified with 1 mg aluminum hydroxide in 200 μL phosphate-buffered saline (PBS) (pH 7.4) as an adjuvant. On days 22, 23, 24, and 25, the mice were challenged by intranasal inhalating 100 μg of OVA in 200 μL PBS once a day. miR-146a mimics (miRBase ID mmu-miR-146a-5p; Invitrogen, Carlsbad, California, USA) and miR-146a inhibitor were intranasally administered at 1 mg/kg body weight every day from day 22 to day 25. For the in vivo TLR activation, 10 μg TLR2 agonist Pam3CSK4 (InvivoGen, San Diego, CA, USA) was intranasally administered every day from day 22 to day 25. Mice were given an equal volume of PBS as the normal control group. Mice were sacrificed 24 h after the last injection. This study was approved by the ethics committee of the Affiliated Wuxi No. 2 People’s Hospital of Nanjing Medical University. All surgeries were performed under anesthesia and efforts were made to minimize the number of animals used in this study.

### Collection of bronchoalveolar lavage fluid (BALF) and peritoneal lavage fluid (PLF)

BALF samples were collected 24 h after the last challenge by 3 perfusions of the whole lung through the trachea with 0.8 ml PBS supplemented with 5 mM EDTA. PLF samples were harvested 4 h after C57BL/6 mice were treated with OVA/alum (i.p.) by intraperitoneally injecting sterile normal saline with 5 mM EDTA.

### Flow cytometry

Cells were stained with corresponding fluorescently labeled antibodies (all from BD Biosciences, Bedford, MA, USA) and assayed or sorted by flow cytometry (FACSAria II cell sorter, BD Biosciences). Eosinophils in BALF samples were characterized as SiglecF^+^CD11c^−^CD11b^+^GR1^lo^. Inflammatory monocytes in PLF were sorted by CD45^+^F4/80^lo^Ly6C^hi^CD11b^+^. CD45 and CD127 antibodies were used to characterize and sort ILC2 from murine lung tissues.

### Cell transfection

Cells were transfected with miR-146a mimics or miR146a inhibitor (500 nM) using the Neon Transfection system (Invitrogen) according to the instructions supplied. The TLR2 agonist Pam3CSK4 (10 μg/mL) was added for 4 h and set as the positive control. Three days later, cells were harvested for further assay.

### Reverse transcription-quantitative polymerase chain reaction (RT-qPCR)

The mRNA levels of IFN-γ, TNF-α, IL-6 and GAPDH in monocytes were detected by qRT-PCR. After total RNA was extracted using TRIzol (Life technologies, Carlsbad, CA, USA), cDNA was synthesized using the Reverse Transcription Kit (Qiagen, Valencia, CA, USA). Primers were designed by Primer 5.0 and sequences (5′ to 3′) were listed as follows: IFN-γ, ACACCACAAGCGACTTGACT (Forward), TCCACATGGCTCATCAACCC (Reverese); TNF-α, GCGGTACTCTGATTCCCTGG (Forward), GTTGGTCTCCGTCCACAGTT (Reverse); IL-6, CCTCTCCGCAAGAGACTTC (Forward), ACCAAACCTCCGACTTGTTGA (Reverse); GAPDH, CCTCAAGATTGTCAGCAAT (Forward), CCATCCACAGTCTTCTGAGT (Reverse). The amplification protocol for the reaction was: preincubation at 95 °C for 4 min, followed by 35 cycles at 95 °C for 30 s, 58 °C for 30 s, and 72 °C for 50 s. The relative mRNA expression levels of targeted genes were calculated using the comparative 2^ΔΔCt^ method. GAPDH was used as the internal standard.

### Lung ILC2 sorting and stimulation

ILC2 were sorted from murine lungs 4 h after OVA-challenge. Single cell suspension was obtained after mincing and digesting of lungs, followed by flow cytometry using CD45 and CD127 surface antibodies. Then sorted ILC2 cells were stimulated by IL-33, TSLP, and IL-7 (10 ng/ml for each) for 5 days, and transfected with miR-146a mimics or miR-146a inhibitor for 3 days.

### Cytometric bead array (CBA)

IL-13 and IL-5 in the culture supernatants of transfected ILC2 cells were detected using the commercial kit BD™ Cytometric Bead Array (CBA, BD Biosciences) according to the manufacture’s protocols.

### ILC2 proliferation assay

To analyze ILC2 proliferation, the single cell suspension was made by digestion with 0.05% trypsin with 0.02% EDTA at 37 °C for 5 min, followed by intracellular staining with fluorescein-conjugated Ki67 antibody (BD Biosciences) for 2 days. LSRII flow cytometry and FlowJo software were used for data analysis.

### ELISA

Samples of serum and BALF were collected 24 h after the last challenge to OVA-induced allergic asthma mice. The concentrations of specific IgE in serum and cytokines, IL-4, IL-5, and IL-13, in BALF were assayed using corresponding ELISA kits (BD Biosciences) according to the manufacturer’s instructions.

### Histological scoring

Lung tissues were fixed in 4% paraformaldehyde for 48 h and embedded in paraffin. Then 7-μm sections were stained with hematoxylin and eosin. The lung inflammation was scored from 0 to 4 as follows: 0 for normal; 1 for few cells; 2 for a ring of inflammatory cells one cell layer deep; 3 for a ring of inflammatory cells two to four cells deep; and 4, a ring of inflammatory cells of more than four cells deep.

### Statistical analysis

Data are presented as mean ± standard deviation (SD) and the statistical significance was evaluated using one-way analysis of variance (ANOVA) and a Tukey’s post hoc test. *P* < 0.05 was considered statistically significant difference.

## Results

### Modeling OVA-induced allergic asthma mice and the effects of miR146a-mimic and miR146a-inhibitor

**Fig. 1:**
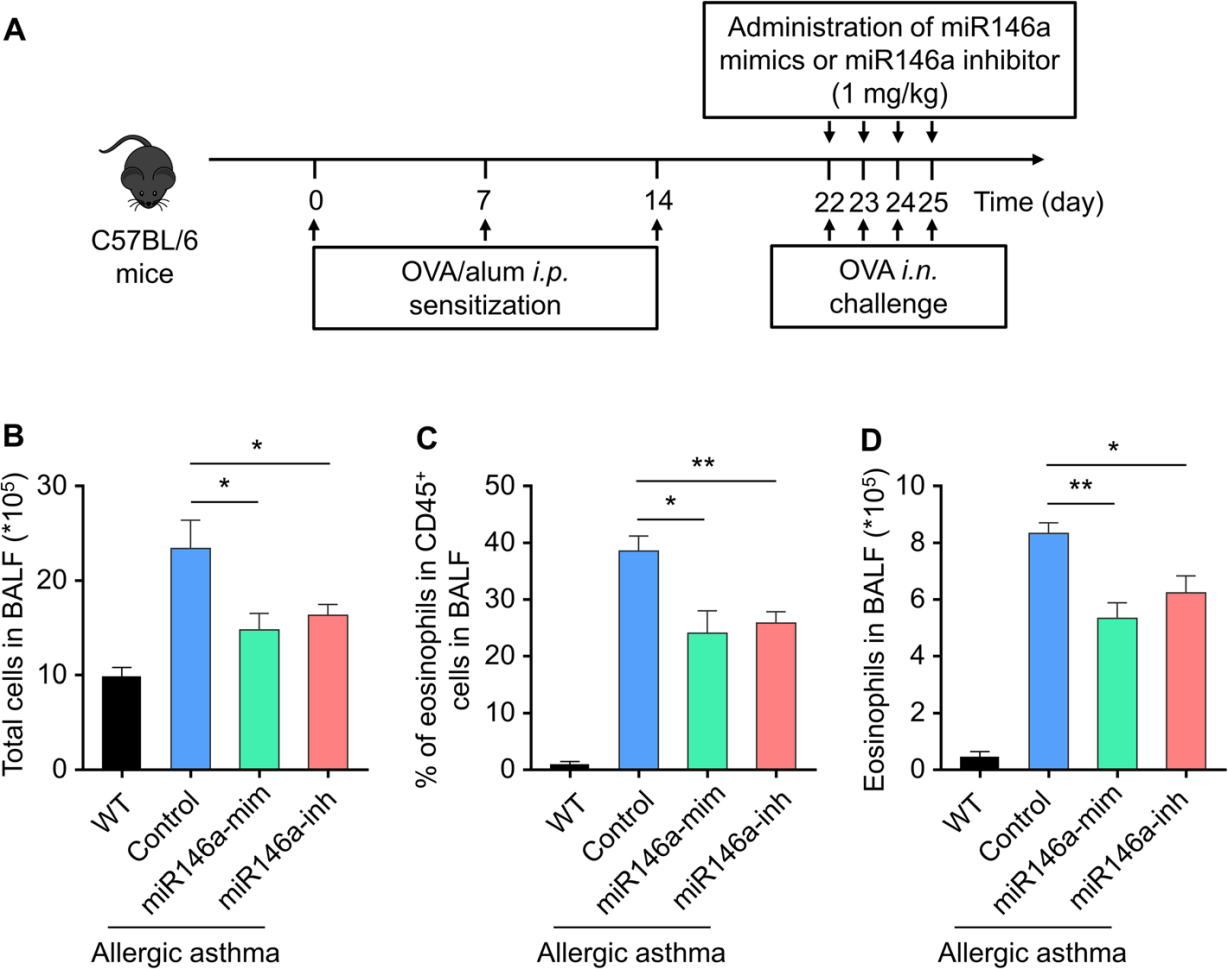
Therapeutic efficacy of miR-146a mimics and miR-146a inhibitor against OVA-induced allergic asthma. **a** Schematic diagram of the experimental protocol. Twenty-four hours after the last challenge, cells collected from BALF were analyzed. **b** Total cells in BALF. **c** Percentage of eosinophils in CD45+ cells in BALF. (D) Eosinophils in BALF. Data represent means ± SD, *n* = 6, * *p* < 0.5; ** *p* < 0.01, ***p < 0.001

Ovalbumin was used to induce mice with allergic asthma. Mice were sensitized by intraperitoneal injection with OVA from day 0–14, and challenged by intranasal inhalations with OVA from day 22 to day 25 once a day (Fig. 1a). To investigate the histological changes in the lung of OVA-challenged mice, H&E staining was performed. Mice showed significant lung inflammation and injury after OVA treatment (Fig. S1A), and scoring of inflammation by pathological evaluation of lung sections numerically illustrated the higher inflammatory cell infiltration OVA-challenged lung than the wild type group (Fig. S1B), indicating that allergic asthma was successfully reproduced in mice by means of OVA challenge in this study. Next, we explored whether miR-146a could influence OVA-induced allergic asthma. It was found that both miR-146a mimics and inhibitor remarkably decreased the abnormal increase of total cells in allergic asthma BALF samples (Fig. 1b). The reactive increase of eosinophils is one of the main characteristics in allergic asthma, which was also observed in the murine model in this study (Fig. 1c and d), further indicating the successful recapitulation of this disease experimentally. Convincingly, miR-146a mimics and inhibitor remarkably rectified the increased BALF eosinophils in asthma mice (Fig. 1c and d). Besides, miRNA mimics control or inhibitor control was administered to asthma mice following the similar experimental diagram and found neither of them influenced the numerical data of total cells (Fig. S2B) as well as eosinophils (Fig. S2C and S2D) in BALF. These results co-indicated that miR146a-mimics and miR146a-inhibitor can successfully decrease eosinophils in OVA-induced allergic asthma mice.

### Effects of miR146a-mimic and miR146a-inhibitor on TLR2 signaling in monocyte

**Fig. 2:**
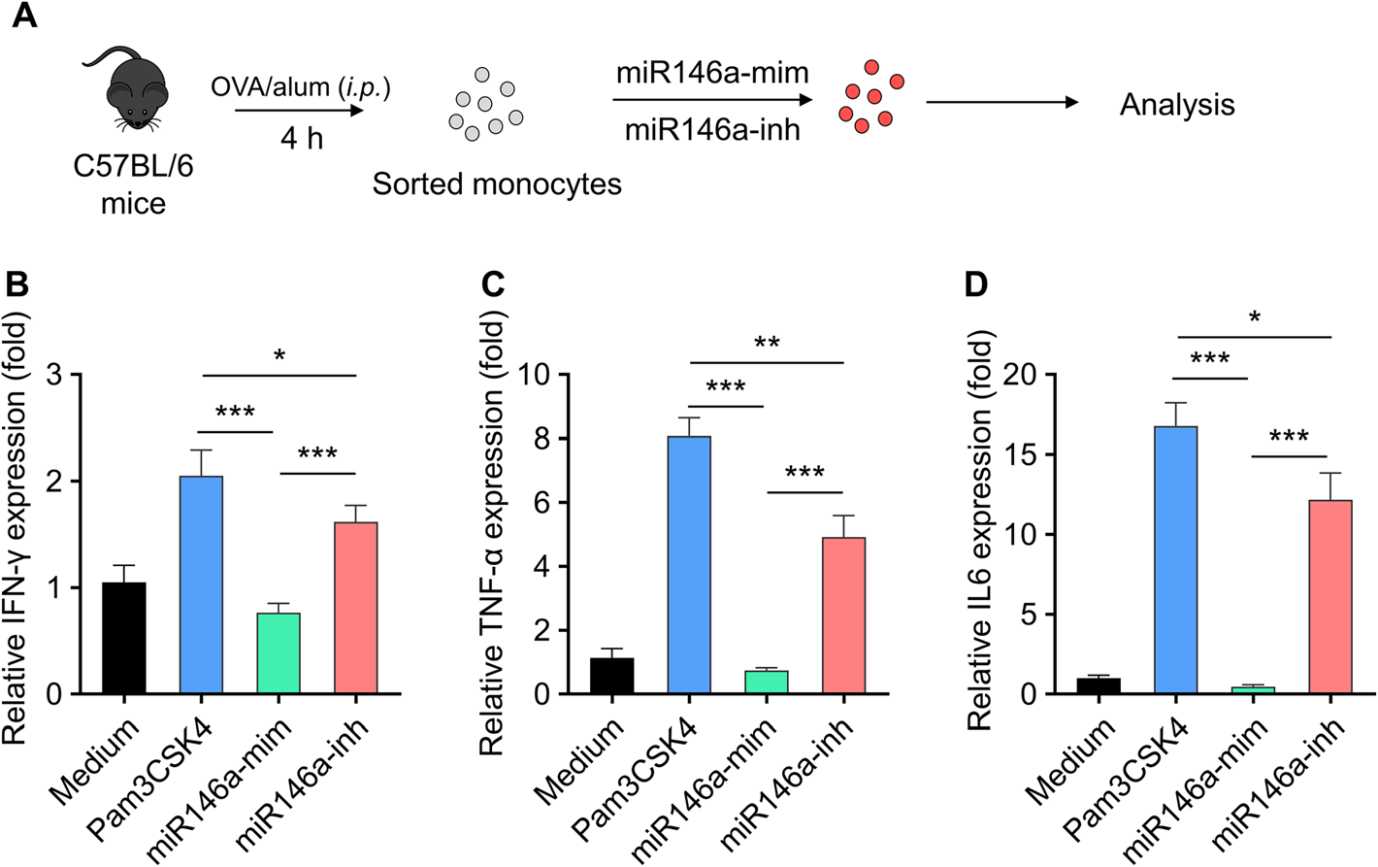
Effect of miR-146a mimics and miR146a inhibitor on activation of TLR2 signaling in monocyte. **a** C57BL/6 mice were treated with OVA/alum (i.p.), and PLF (peritoneal lavage fluids) were harvested 4 h after treatment to purify inflammatory monocytes by sorting. Sorted monocytes were transfected with miR-146a mimics or miR146a inhibitor (500 nM) for 3 days, the TLR2 agonist was set as positive control. Expressions of Th1-type cytokines, including IFN-γ **b** TNF-α **c** and IL-6 **d** were analyzed by real-time PCR. Data represent means ± SD, *n* = 3, ***p < 0.001. The in vitro experiments were repeated three times

To evaluate the effect of miR-146a on monocyte, peritoneal lavage fluids were collected from OVA/alum (*i.p.*) treated mice to purify inflammatory monocytes, which were used to be transfected with miR146a-mimic and miR146a-inhibitor for 3 days (Fig. 2a). In order to detect whether the TLR2 signaling pathway can be influenced after miR146a-mimic or miR146a-inhibitor administration in OVA-treated inflammation monocyte, the expression of cytokines were analyzed by real-time PCR, including IFN-γ (Fig. 2b), TNF-α (Fig. 2c) and IL-6 (Fig. 2d), with TLR2 agonist Pam3CSK4 as the positive control. Similarly, transfection of miRNA mimics control or inhibitor control (Fig. S3A) did not affect the expression of the above cytokines (Fig. S3B-S3D). LPS, a TLR4 agonist, served as a control to validate the therapeutic effect of miR-146a mimic in the OVA-induced allergic asthma model. miR146a-mimic could significantly decrease the expression of IFN-γ, TNF-α and IL-6 compared with positive group, while miR146a-inhibitor administration can upregulate the expression of these cytokines (Fig. 2). Furthermore, both LPS and miR-146a mimic treatment significantly reduced the expressions of Th1-type cytokines (Fig. S4A-D). Therefore, the treatment of miR146a-mimic inhibits TLR2 pathway, while the miR146a-inhibitor acts as Pam3CSK4 to activate TLR2 signaling pathway.

### Effect of miR146a-mimic and miR146a-inhibitor on the function and proliferation of ILC2

**Fig. 3:**
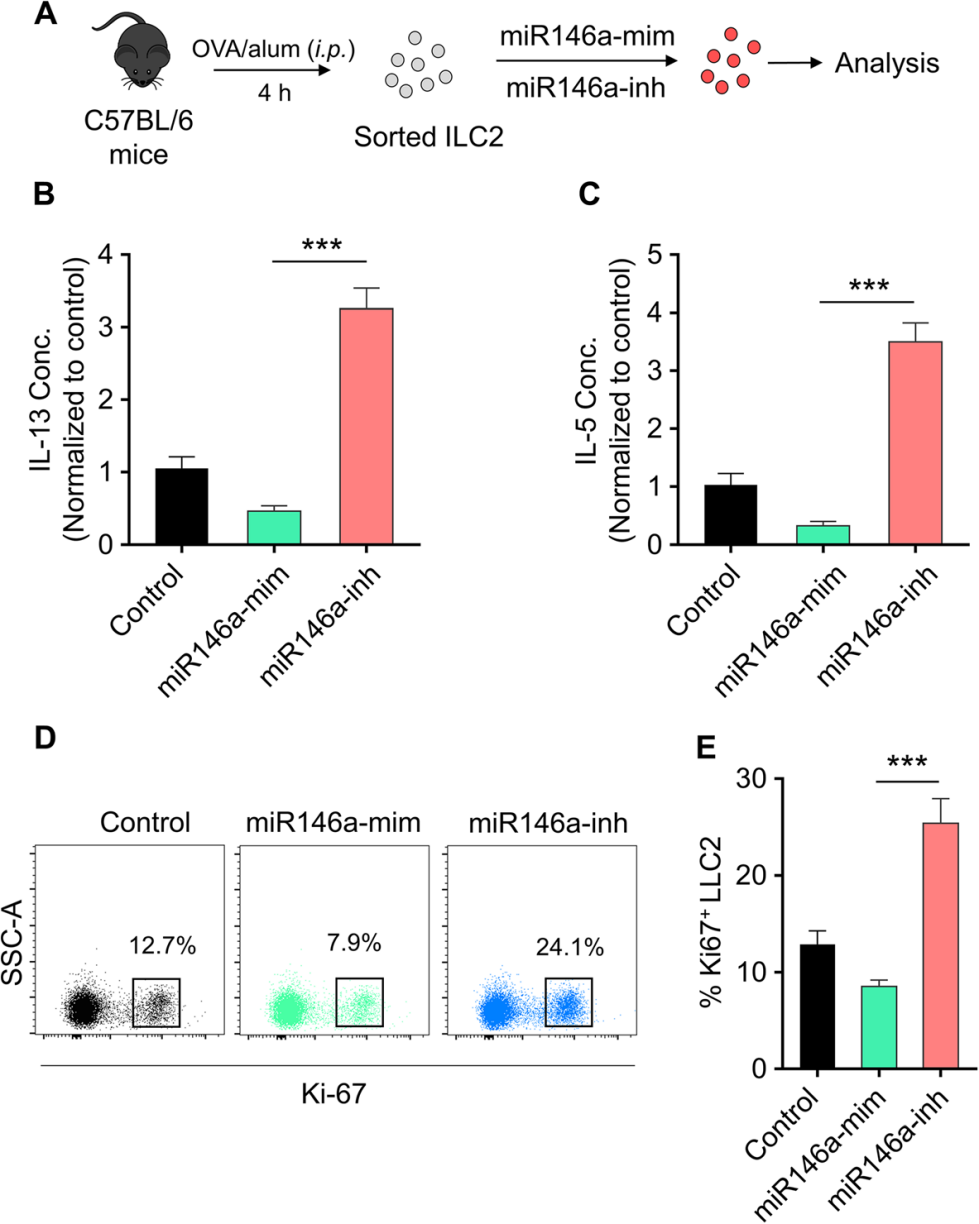
Effect of miR-146a mimic and miR146a inhibitor on the function and proliferation of ILC2. **a** Schematic diagram of the in vitro culture: ILC2 were sorted from PLF of OVA-challenged mice and cultured with IL-33, TSLP, and IL-7 (10 ng/ml each) for 5 days then transfected with miR-146a mimics or miR-146a inhibitor for 3 days. IL-13 (**b**) and IL-5 (**c**) were measured by cytometric bead array (CBA) in supernatants. ILC2 proliferation were analyzed by ki-67 staining. The dot plot of FACS (**d**) and statistical analysis (**e**) were shown. Data represent means ± SD, * p < 0.5; ** p < 0.01, ***p < 0.001

Previous study found that miR-146a mimics could inhibit ILC2 proliferation. In this study, to detect the function of miR-146a, ILC2 was first sorted from PLF of OVA-challenged mice. Then ILC2 were stimulated with IL-33, TSLP and IL-7 for 5 days followed by transfection with miR146a-mimics or miR146a-inhibitor (Fig. 3a). IL-5 and IL-13 secretion is important for ILC2 to exert its effector function. The results showed that, miR146a-mimic inhibited their secretion, while the miR146a-inhibitor promoted the secretion (Fig. 3b and c). To investigate its intrinsic mechanism, we observed whether miR146a-mimic administration in vitro affected ILC2 cell proliferation. Flow cytometry analysis indicated that, compared with the control group, there were less Ki67^+^ ILC2 in miR146a-mimic group, while more Ki67^+^ ILC2 in miR146a-inhibitor group, almost twice as large as the control (Fig. 3d and e). These results suggest that miR146a-mimic inhibits ILC2 proliferation.

### TLR2 agonist and miR146a mimics efficiently protect mice against OVA-induced allergic asthma

**Fig. 4:**
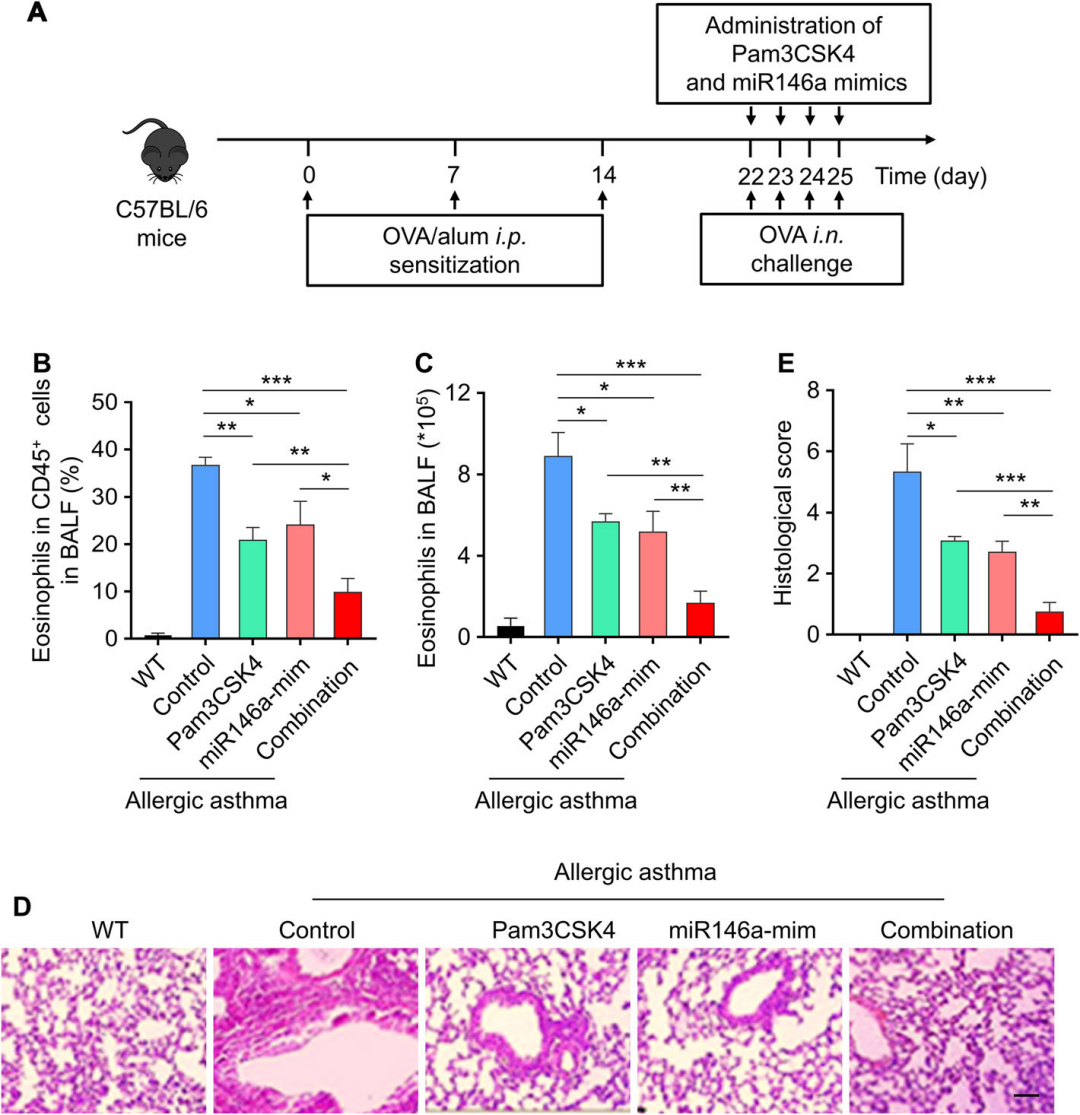
Simultaneous administration of TLR2 agonist and miR146a mimics efficiently protect mice against OVA-induced allergic asthma. **a** Schematic diagram of the experimental protocol. The doses of Pam3CSK4 and miR146a mimics were 0.1 mg/kg and 1 mg/kg, respectively. **b** Eosinophils in CD45+ cells in BALF. **c** Eosinophils in BALF. **d** Histologic sections of lungs from each group were analyzed by H&E. Pictures show representative samples of 6 mice/group. Scale bar = 100 μm. **e** Histological scores for assessment of lung injury were shown. Data represent means ± SD. WT, wide type mice without OVA-induced allergic asthma. *n* = 6, *p < 0.05, **p < 0.005, ***p < 0.001

From the above results, it is known that miR146a-mimic plays an important role in promoting the activation of TLR2 as its agonist. So, does the combination of miR146a- mimic and Pam3CSK4 affect eosinophils in BALF? To explore this issue, we administered miR146a-mimic and Pam3CSK4 from day 22–25 in OVA-induced allergic asthma mice model (Fig. 4a and S5A), then counted the cells in wild-type group, control group, Pam3CSK4 group, miR146a-mimic group and combination group. Eosinophils in CD45^+^ cells and eosinophils in BALF significantly reduced in both separate groups (Pam3CSK4 group and miR146a-mimic group) and combined group (Combination group) (Fig. 4b and c). What’s more, eosinophils in CD45^+^ cells has significantly decreased in miR146a-inhibitor combination group compared to Pam3csk4 combination group (Fig. S5B), and we found that these two combinations evidently decreased the increased eosinophils in asthma BALF (Fig. S5C). H&E was used to assess the injury of lung in these five groups, both separate groups (Pam3CSK4 group and miR146a-mimic group) and combined group (Combination group) decreased the injury compared with control group (Fig. 4d and e). It’s suggested that TLR2 agonist and miR146a mimics can protect against OVA-induced allergic asthma efficiently.

### Simultaneous administration of TLR2 agonist and miR146a mimics attenuates OVA-induced allergic asthma by increasing Th1 cytokines

**Fig. 5:**
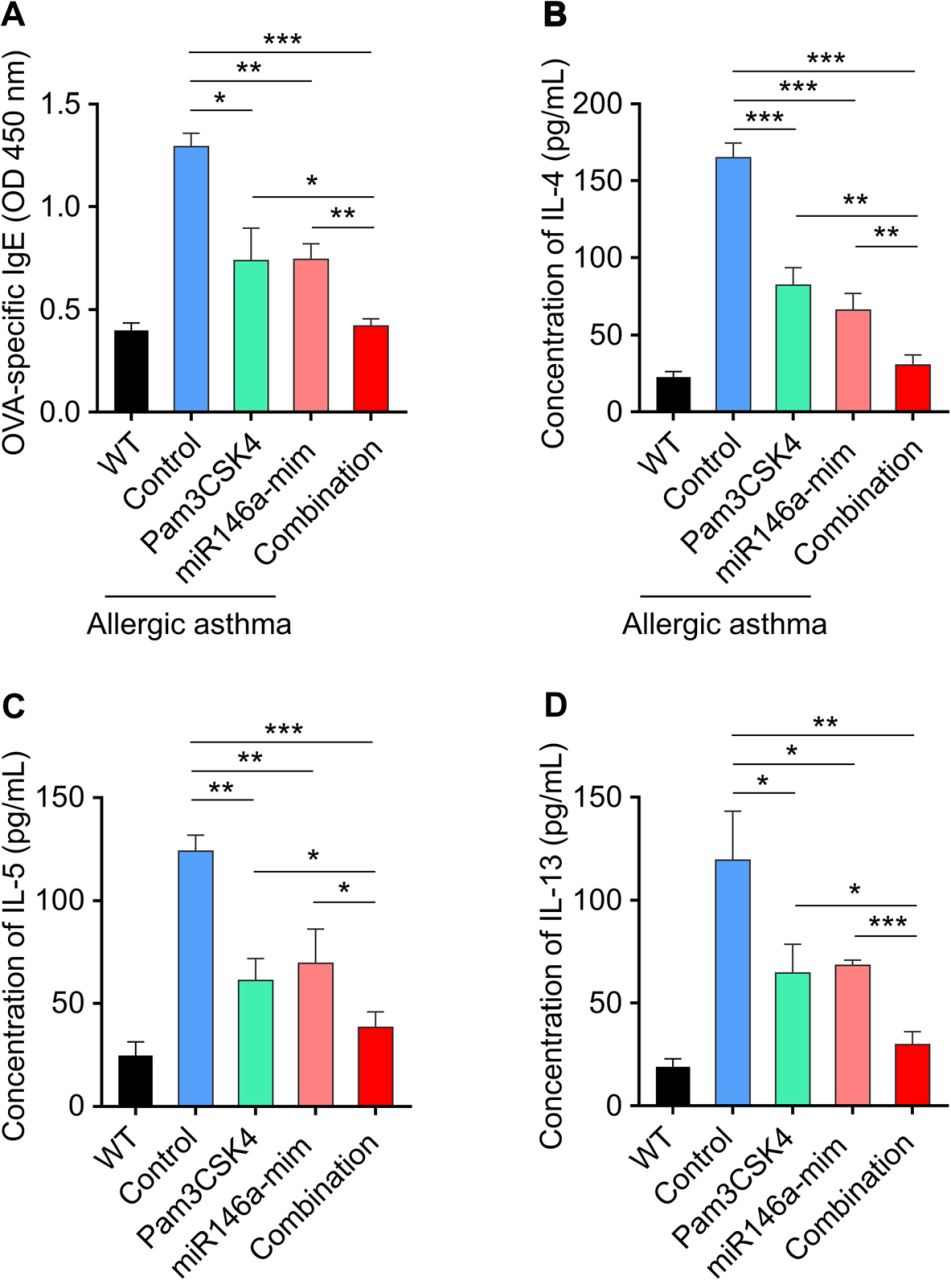
Simultaneous administration of TLR2 agonist and miR146a mimics attenuates OVA-induced allergic asthma by increasing Th2 cytokines. The levels of OVA-specific IgE in serum (**a**), IL-4 (**b**), IL-5 (**c**), and IL-13 (**d**) in BALF. Serum and BALF were collected 24 h after the last challenge. The levels of OVA-specific IgE and these cytokines were quantified by ELISA. Data represent means ± SD. WT, wide type mice without OVA-induced allergic asthma. n = 6, *p < 0.05, **p < 0.005, ***p < 0.001

To explore how TLR2 agonist and miR146a-mimic attenuate OVA-induced allergic asthma, we considered Th1 cytokines, including IgE, IL-4, IL-5 and IL-13 (Fig. 5a-d), might be involved. The levels of OVA-specific IgE in serum, IL-4, IL-5 and IL-13 in BALF were quantified by ELISA. Pam3CSK4 group, miR146a-mimic group and combination group all decreased the level of Th1 cytokines. These results indicated that TLR2 agonist and miR146a-mimic might attenuate OVA-induced allergic asthma through downregulating the level of Th1 cytokines.

## Discussion

Asthma is one of the most common chronic diseases, an immune response identified by airway stenosis or inflammation, which affects around 300 million people worldwide (Chung 2015; Cohn et al. 2004; Galli et al. 2008). Our results about inflammatory cells in BALF are consistent with the previous studies that asthma is caused by a variety of inflammatory cells such as eosinophils, and these cells decreased after miR146a-mimic administration. We examined the biological effect of miRNA mimics controls and miRNA inhibitor control in ovalbumin-induced asthma mice model and confirmed that negative miRNA controls do not exhibit ameliorative effect on asthma mice, as well as do not affect the cytokines production of sorted monocytes. miR-146a mimic and miR-146a inhibitor attenuate ovalbumin-induced asthma via different signal pathways. miR-146a mimics attenuate allergic airway inflammation by impacting ILC2 via IRAK1/TRAF6 pathways, while miR-146a inhibitors attenuate asthma via activating TLR2-signaling pathways in inflammatory monocytes. In our experiments, we also observed the therapeutic efficacy of miR-146a mimics and miR-146a inhibitor against OVA-induced allergic asthma (including decreased eosinophils in BALF), which is consistent to previous reports.

Previous studies found that miR-146a administration in vivo alleviated the symptoms of ovalbumin (OVA)-induced asthma in mice through the IL-33 pathway (Li et al. 2014; Liu et al. 2017; Lyu et al. 2019). MiR-146 includes two members, miR-146a and miR-146b, which were reported as negative regulators of cellular inflammatory cytokines in monocyte (Etikala et al. 2015). Therefore, we isolated the monocytes in the peritoneal lavage fluid of OVA-sensitized mice, and cells transfected with miR-146a inhibitor upregulate the expression of cytokines in TLR2 signaling pathway. Regarding the combination of miR146a-inhibitor and miR146a-mimic, we also observed efficient anti-asthma effect, but the combination of miR146a mimic and Pam3CSK4 (TLR2 agonist) was the most efficient.

ILC2 is differentiated from ILC3 in response to activation of TLR2 and produces IL-5 and IL-13 (Alderton 2012; Wills-Karp 2004), which plays vital role in initiating and promoting immune response among asthma patients, and leads to the accumulation of eosinophils in BALF (Izuhara 2017; Lloyd and Hessel 2010). IL-5 and IL-13 are cytokines for the survival and maturation of eosinophils, which were detected to be significantly increased after treatment with miR146a-inhibitor in this study.

Inspired by the findings in previous studies (Huang et al. 2018; Zhang et al. 2019), we propose that a combination of TLR2 agonists and miR-146a mimics may have a better response to OVA-induced allergic asthma. Experiments in vivo in our study have also proved that the combination of these two can achieve better therapeutic effect than using one alone.

Nowadays, little is known about the mechanism of allergic asthma which greatly hinders the development for efficient therapeutic strategies, and it is urgent to make a breakthrough (Papi et al. 2018; Yao et al. 2012). Our research found that miR-146a may regulate the activation of TLR2 signaling pathway, which is mediated by Th1 cytokines. Therefore, our study provides a theoretical basis for the treatment of asthma.

## Conclusions

In the experiment of OVA-induced allergic asthma model, it was found that miR-146a is an important regulator, which can regulate TLR2 pathway mediated by Th1 cytokines, thereby relieving symptoms of asthma.

## Supplementary information

**Additional file 1: Figure S1.** OVA induced inflammation and lung injuries. (A) H&E staining was performed to evaluate OVA-induced inflammation and lung injury in asthma. (B) Scoring of inflammation was conducted through pathological evaluation of the inflammatory cell infiltrate in lung sections. Data represent means ± SD. ***p < 0.001. **Figure S2.** (A) Schematic diagram of the experimental protocol. Twenty-four hours after the last challenge, cells collected from BALF were analyzed. (B) Total cells in BALF. (C) Percentage of eosinophils in CD45+ cells in BALF. (D) Eosinophils in BALF. Data represent means ± SD, ns, no significantly difference. **Figure S3.** (A) C57BL/6 mice were treated with OVA/alum (*i.p*.), and PLF (peritoneal lavage fluids) were harvested 4 h after treatment to purify inflammatory monocytes by sorting. Sorted monocytes were transfected with miRNA mimics control or miRNA inhibitor control (500 nM) for 3 days. Expressions of Th1-type cytokines, including IFN-γ (B) TNF-α (C) and IL-6 (D) were analyzed by real-time PCR. Data represent means ± SD, ns, no significantly difference. **Figure S4.** (**A**) C57BL/6 mice were treated with OVA/alum (*i.p*.), and PLF (peritoneal lavage fluids) were harvested 4 h after treatment to purify inflammatory monocytes by sorting. Sorted monocytes were transfected with miR146a-mim (500 nM) or treated with LPS (10 ng/mL) for 3 days. Expressions of Th1-type cytokines, including IFN-γ (B) TNF-α (C) and IL-6 (D) were analyzed by real-time PCR. Data represent means ± SD, *p < 0.05, **p < 0.01. **Figure S5**. (**A**) Schematic diagram of the experimental protocol. The dose of Pam3CSK4 was 0.1 mg/kg, while the doses of miR146a mimics and miR146a inhibitor were 1 mg/kg, respectively. (B) Percentage of eosinophils in CD45^+^ cells in BALF. (C) Eosinophils in BALF. Data represent means ± SD, *p < 0.05.

## Data Availability

Data could be obtained upon request to the corresponding author.
