# Supplementary material for: Combination of TLR agonist and miR146a mimics attenuates ovalbumin-induced asthma
Source: Mol Med. 2020 Jun 29;26:65. doi: 10.1186/s10020-020-00191-1 (PMC7325265; doi:10.1186/s10020-020-00191-1)
Supplement: Supplementary file 1 — Additional file 1: Figure S1. OVA induced inflammation and lung injuries. (A) H&E staining was performed to evaluate OVA-induced inflammation and lung injury in asthma. (B) Scoring of inflammation was conducted through pathological evaluation of the inflammatory cell infiltrate in lung sections. Data represent means ± SD. ***p < 0.001. Figure S2. (A) Schematic diagram of the experimental protocol. Twenty-four hours after the last challenge, cells collected from BALF were analyzed. (B) Total cells in BALF. (C) Percentage of eosinophils in CD45+ cells in BALF. (D) Eosinophils in BALF. Data represent means ± SD, ns, no significantly difference. Figure S3. (A) C57BL/6 mice were treated with OVA/alum (i.p.), and PLF (peritoneal lavage fluids) were harvested 4 h after treatment to purify inflammatory monocytes by sorting. Sorted monocytes were transfected with miRNA mimics control or miRNA inhibitor control (500 nM) for 3 days. Expressions of Th1-type cytokines, including IFN-γ (B) TNF-α (C) and IL-6 (D) were analyzed by real-time PCR. Data represent means ± SD, ns, no significantly difference. Figure S4. (A) C57BL/6 mice were treated with OVA/alum (i.p.), and PLF (peritoneal lavage fluids) were harvested 4 h after treatment to purify inflammatory monocytes by sorting. Sorted monocytes were transfected with miR146a-mim (500 nM) or treated with LPS (10 ng/mL) for 3 days. Expressions of Th1-type cytokines, including IFN-γ (B) TNF-α (C) and IL-6 (D) were analyzed by real-time PCR. Data represent means ± SD, *p < 0.05, **p < 0.01. Figure S5. (A) Schematic diagram of the experimental protocol. The dose of Pam3CSK4 was 0.1 mg/kg, while the doses of miR146a mimics and miR146a inhibitor were 1 mg/kg, respectively. (B) Percentage of eosinophils in CD45+ cells in BALF. (C) Eosinophils in BALF. Data represent means ± SD, *p < 0.05. [file 10020_2020_191_MOESM1_ESM.docx]

**Supplemental Information**

**
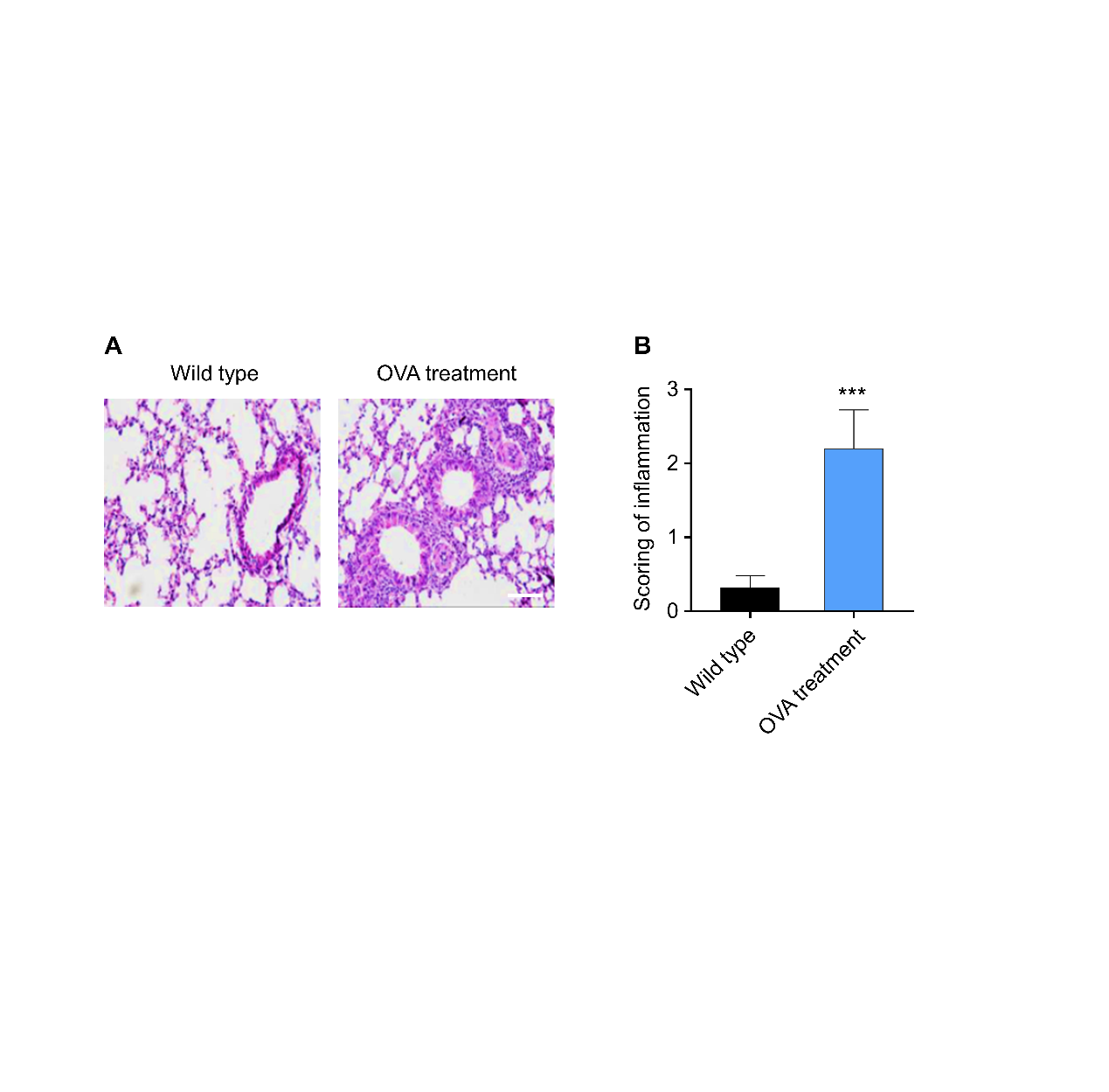
**

**Figure S1.** OVA induced inflammation and lung injuries. (**A**) H&E staining was performed to evaluate OVA-induced inflammation and lung injury in asthma. (**B**) Scoring of inflammation was conducted through pathological evaluation of the inflammatory cell infiltrate in lung sections. Data represent means ± SD. ****p* < 0.001.


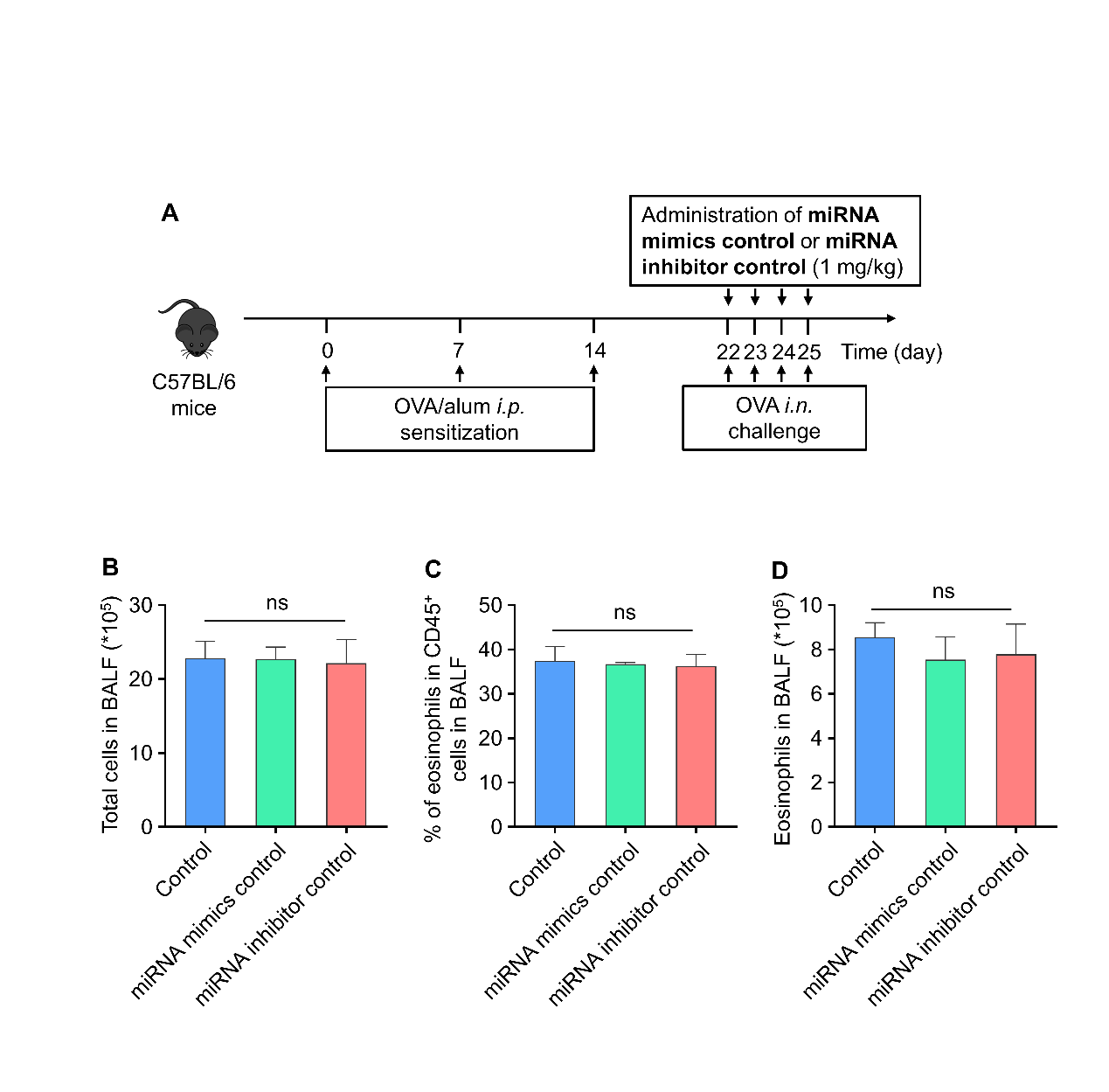


**Figure S2.** (**A**) Schematic diagram of the experimental protocol. Twenty-four hours after the last challenge, cells collected from BALF were analyzed. (**B**) Total cells in BALF. (**C**) Percentage of eosinophils in CD45+ cells in BALF. (**D**) Eosinophils in BALF. Data represent means ± SD, ns, no significantly difference.


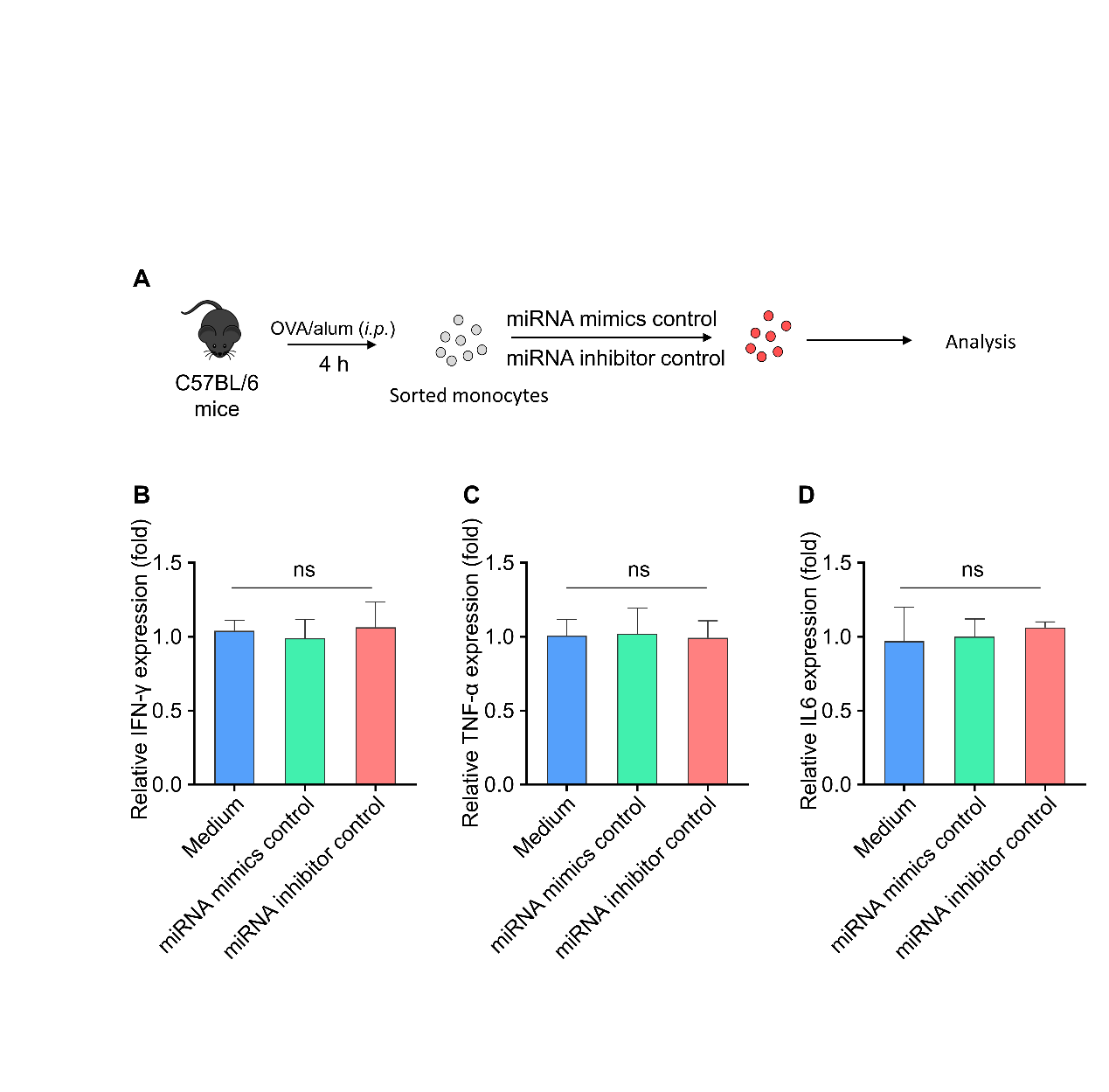


**Figure S3.** (**A**) C57BL/6 mice were treated with OVA/alum (*i.p*.), and PLF ([peritoneal](javascript:;) [lavage](javascript:;) [fluids](javascript:;)) were harvested 4 h after treatment to purify inflammatory monocytes by sorting. Sorted monocytes were transfected with miRNA mimics control or miRNA inhibitor control (500 nM) for 3 days. Expressions of Th1-type cytokines, including IFN-γ (**B**) TNF-α (**C**) and IL-6 (**D**) were analyzed by real-time PCR. Data represent means ± SD, ns, no significantly difference.


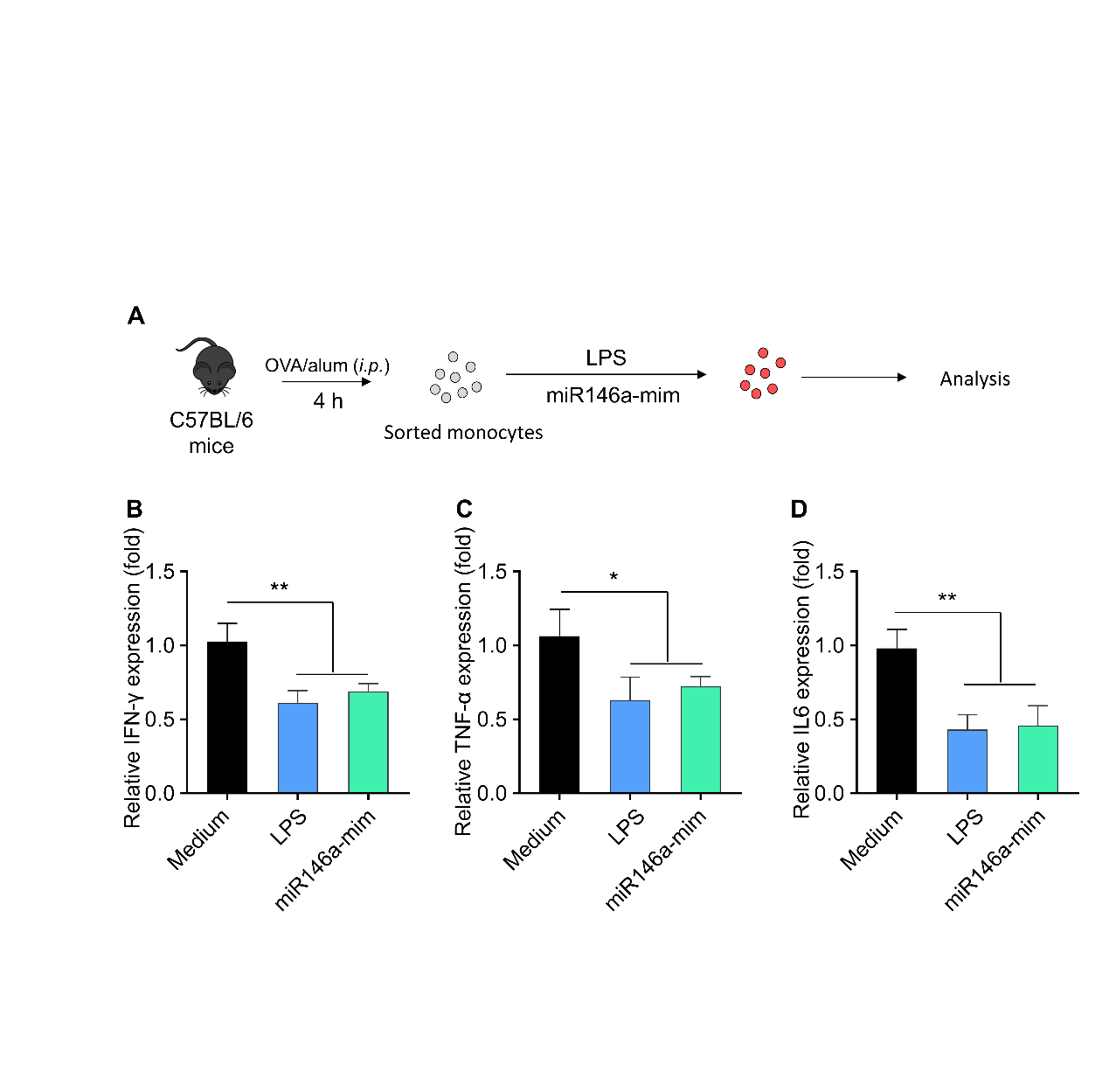


**Figure S4.** (**A**) C57BL/6 mice were treated with OVA/alum (*i.p*.), and PLF ([peritoneal](javascript:;) [lavage](javascript:;) [fluids](javascript:;)) were harvested 4 h after treatment to purify inflammatory monocytes by sorting. Sorted monocytes were transfected with miR146a-mim (500 nM) or treated with LPS (10 ng/mL) for 3 days. Expressions of Th1-type cytokines, including IFN-γ (**B**) TNF-α (**C**) and IL-6 (**D**) were analyzed by real-time PCR. Data represent means ± SD, **p* < 0.05, ***p* < 0.01.


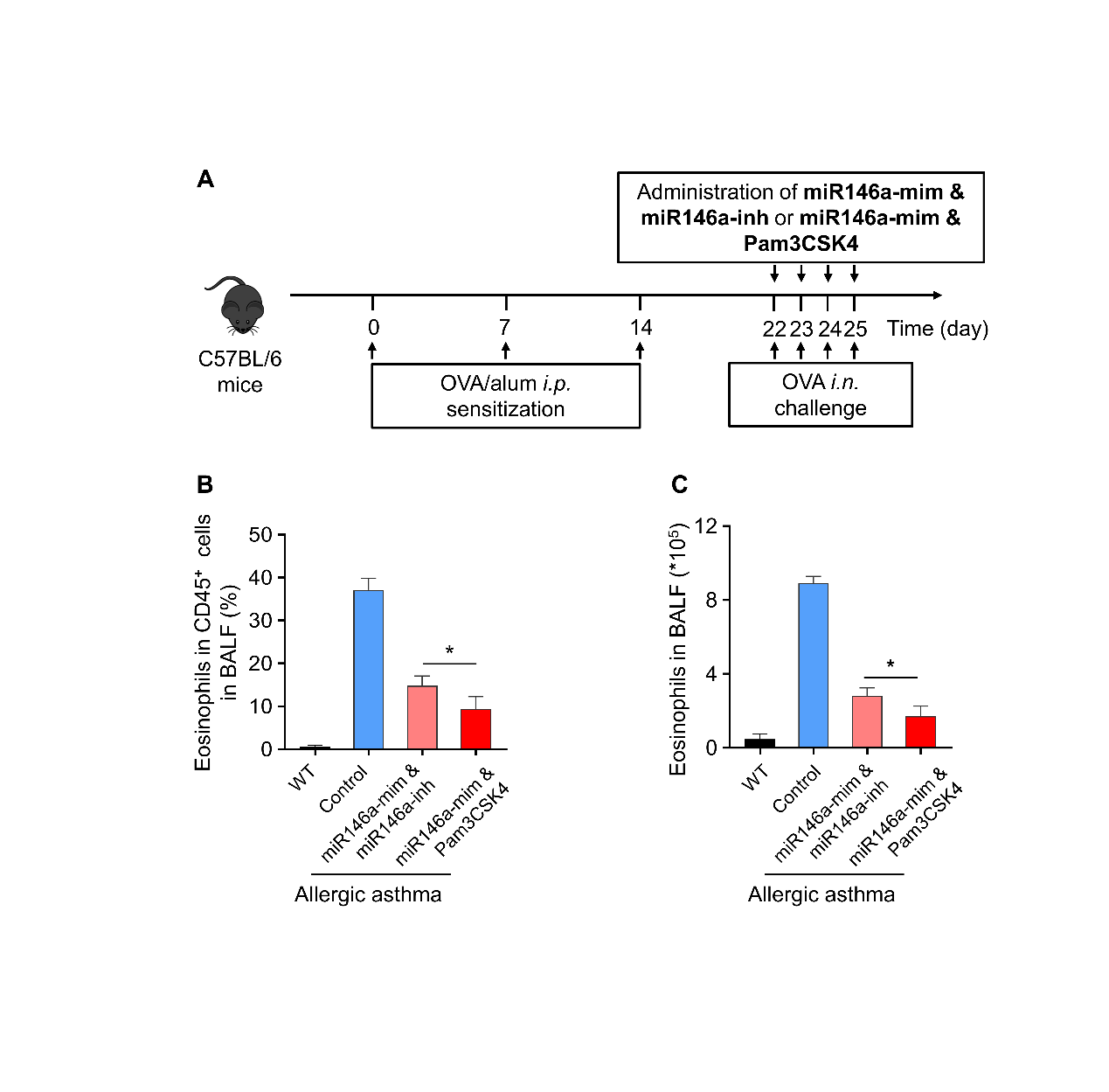


**Figure S5**. (**A**) Schematic diagram of the experimental protocol. The dose of Pam3CSK4 was 0.1 mg/kg, while the doses of miR146a mimics and miR146a inhibitor were 1 mg/kg, respectively. (**B**) Percentage of eosinophils in CD45^+^ cells in BALF. (**C**) Eosinophils in BALF. Data represent means ± SD, **p* < 0.05.
